# Faciobrachial Myoclonus as the Presenting Manifestation of Diabetic Keto-Acidosis

**DOI:** 10.5334/tohm.605

**Published:** 2021-03-02

**Authors:** Subhankar Chatterjee, Ritwik Ghosh, Rinky Kumari, Umesh Kumar Ojha, Julián Benito-León, Souvik Dubey

**Affiliations:** 1 Department of General Medicine, Patliputra Medical College & Hospital, Dhanbad, India; 2 Department of General Medicine, Burdwan Medical College & Hospital, Burdwan, India; 3 Department of Neurology, University Hospital “12 de Octubre”, Madrid, Spain; 4 Centro de Investigación Biomédica en Red sobre Enfermedades Neurodegenerativas (CIBERNED), Madrid, Spain; 5 Department of Medicine, Complutense University, Madrid, Spain; 6 Department of Neuromedicine, Bangur Institute of Neurosciences, Institute of Neurosciences, Institute of Postgraduate Medical Education and Research & SSKM Hospital, Kolkata, India

**Keywords:** Myoclonus, Faciobrachial dystonic seizures, Hyperglycemia, Diabetic ketoacidosis, Movement disorder

## Abstract

**Background:**

Chorea and ballism are well-recognized acute potentially reversible movement disorders as the presenting manifestation of non-ketotic hyperglycemic states among older type-2 diabetics. Myoclonus as the form of presentation of diabetic keto-acidosis (DKA) in previously undiagnosed type-1 diabetic has never been reported before.

**Case report:**

We herein report the case of a 36-year-old previously healthy patient who presented with acute onset incessant faciobrachial myoclonus for 10 days. The patient was found to be suffering from DKA and eventually diagnosed as type-1 diabetes mellitus. Myoclonus disappeared with achieving euglycemia and did not recur.

**Discussion:**

Apart from expanding the spectrum of acute movement disorder among diabetics, this case reiterates the importance of rapid bedside measurement of capillary blood glucose in all patients presenting with acute onset abnormal movements irrespective of their past glycemic status. This simple yet life-saving approach can clinch the diagnosis at the earliest and thus will avoid costly investigations and mismanagement.

## Introduction

The horizon of movement disorders in diabetes mellitus is expanding. While chorea and ballism are well-recognized acute potentially reversible movement disorders as the presenting manifestation of non-ketotic hyperglycemic states among older type-2 diabetics [1], different types of myoclonus are also being recently reported in this context [2]. We herein report the case of a 36-year-old previously healthy patient who presented with acute onset incessant faciobrachial myoclonus for 10 days. The patient was found to be suffering from diabetic keto-acidosis (DKA) and eventually diagnosed as type-1 diabetes mellitus (T1DM). This case highlights the importance of precise identification of the semiology of abnormal movements and the obligatory measurement of capillary blood glucose (CBG) at bedside in all patients with acute onset movement disorders.

## Case report

A 36-year-old previously healthy man presented to the emergency room with acute onset abnormal movements involving face and upper limbs for 10 days and gradually developing altered sensorium for last 6 hours. He was hospitalized in a rural health facility where his clinical picture was initially considered as due to prolonged seizure activity for which he received multiple anti-epileptic drugs. As he was progressively deteriorating, he was referred to a tertiary center. As per the caregiver’s description and medical records received from the previous hospital, he was having abnormal, involuntary, rapid, brief and jerky movements involving face and upper limbs (predominantly right sided). Although there was no history of headache, fever, vomiting, and any focal weakness, the patient had significant involuntary weight loss in last 4 months. Family history and drug history were non-contributory.

Clinical examination revealed the patient was drowsy, dehydrated, cachectic, afebrile, tachycardic (130/min), normotensive (100/60 mmHg) and tachypneic (36/min). He had faciobrachial myoclonic jerks with asymmetric involvement of right upper limb more than left upper limb (distal more than proximal), with tonic neck deviation to left side withoutany abnormal movement of bilateral lower limbs (***Video 1***). The myoclonic jerks were not associated with any external cues or voluntary action. The tone and deep tendon reflexes of upper limbs could not be assessed due to persistent abnormal involuntary movements. The tone and deep tendon reflexes of lower limbs were normal; the plantar reflexes were flexor. Complete assessment of cranial nerves, sensory, cerebellar and autonomic functions could not be done. There was no sign of meningeal irritation and papilledema.

**Video 1:** Video showing faciobrachial myoclonus with asymmetric involvement of right upper limb more than left upper limb. Notice the tonic neck deviation to left side.

Acute onset multifocal myoclonus associated with encephalopathy with a background of addiction and recent onset progressive involuntary weight loss had narrowed the differential diagnoses to toxic-metabolic and neuro-infectious etiologies. Bedside CBG was measured immediately and was found to be 695 mg/dL (normal, <200). Arterial blood gas (ABG) analysis revealed metabolic acidosis (pH- 7.28 [normal, 7.35–7.45], HCO_3_^-^ -18 mEq/L [normal, 22–26], Na^+^- 117 mEq/L [normal, 135–145] [131 mEq/L when corrected for hyperglycemia], anion gap- 18 mmol/L (normal<12), with normal serum osmolality, pO_2_, pCO_2_, K^+^, and ionic Ca^2+^). Urine examination showed glycosuria (3+) and ketonuria (2+); plasma beta-hydroxy-butyrate level was found to be 3.6mmol/L (normal, <1.5). Thus, a diagnosis of DKA was confirmed. Adequate hydration was ensured with normal saline followed by treatment with continuous intravenous regular insulin (at a rate of 8 IU/h)., Once CBG came down to less than 400 mg/dL, approximately 3 h after therapy, all movements disappeared. His metabolic derangements were completely corrected by next 2 days and CBG was fairly controlled with basal-bolus regimen.

After stabilization he had absolutely normal hemodynamic and hydration status. Detailed neurological and other systemic examinations were within normal limits. His HbA1c was 10.62% (normal, <5.6), fasting and post-prandial blood glucose were 137 mg/dL (normal, <100) and 250 mg/dL (normal, <140) respectively. C peptide level was low [0.1 ng/ml (normal, 0.5–2.7)] with positive anti-glutamic acid decarboxylase (GAD)-65 antibodies that established the diagnosis of T1DM. The patient’s oropharyngeal swab test for SARS-CoV-2, by qualitative real-time reverse-transcriptase–polymerase-chain-reaction assay, was negative.

All other tests of the metabolic panel (complete hemogram, lipid profile, renal, liver, thyroid function tests) were within normal limits. Autoimmune and paraneoplastic encephalitis profile [anti-nuclear antibodies, anti-thyroid antibodies, antibodies against leucine-rich glioma-inactivated 1 (anti-LGl1), contactin-associated protein-like 2 (Caspr2), and N-methyl-D-aspartate receptor (NMDA-R)] were negative. Serological tests for HIV 1&2, and hepatitis B and C were negative. Brain magnetic resonance imaging (MRI), interictal electroencephalogram (EEG), and cerebrospinal fluid analysis were normal. The rest of his hospital stay was uneventful and did not have any recurrence of involuntary movements during a 2-month follow-up.

## Discussion

The most important aspect of this case was to correctly identify the semiology of the abnormal hyperkinetic movements at first place. From the description of the attendant and our clinical observation, multifocal positive myoclonus was at the top of the list of differential diagnoses [3]. The other differential diagnoses are described in ***Table 1***.

**Table 1:** Differential diagnoses of faciobrachial myoclonus.


DIFFERENTIAL DIAGNOSES	POINTS AGAINST THIS DIAGNOSIS

Epilepsia partialis continua with secondary generalization [3,4]	No abnormal movements of lower limbs

Faciobrachial dystonic seizures [5,6,7]	a) Faciobrachial dystonic seizures is characterized by unilateral short-lived dystonic posturing of face and unilateral upper arm that lifts the affected arm from bed in most cases (movements in our case was clearly bilateral and synchronous with no arm lifting from the bed); b) No behavioral, psychiatric or cognitive impairment which is almost always associated with the cases of faciobrachial dystonic seizures in autoimmune encephalitis; c) Most remarkably, all the abnormal movements and encephalopathy were abolished by correction of hyperglycemia alone without any immunomodulatory therapy, which also refuted the diagnosis of anti-GAD associated myoclonus; d) No imaging abnormality in fronto-ponto-basal ganglia and cerebello-thalamo-cortical networks; and e) Negative auto-antibody profile, including anti-LGI-1 and Caspr-2.

Wernicke’s encephalopathy with myoclonus	Serum thiamine level was normal and brain MRI revealed no abnormality

Anti-GAD antibody associated limb myoclonus with encephalopathy [17]	Correction of hyperglycemia alone abated myoclonus obviating any need for steroids or immunotherapy

Sporadic Creutzfeldt-Jakob disease	Normal MRI brain and cerebrospinal fluid


As the myoclonus followed by encephalopathy was of acute onset without any other neurological manifestations (i.e. seizures or dystonia) in a previously healthy adult patient, it was initially considered as functional myoclonus [3,8]. While unfurling the cause, metabolic derangements were readily identified in the form of ketotic hyperglycemia and mild hyponatremia. The cause of the myoclonus was hyperglycemia as myoclonus ceased just after its correction (much earlier than after achieving eunatremia and normal pH) [8]. Possible coexisting secondary causes were excluded by appropriate tests and historical analysis [8].

Myoclonus among diabetic patients may generally occur in the setting of uremia, resulting from diabetic nephropathy [9], drug-toxicity [10], or in relation with some rare syndromes [11]. Dubey et al [2] reported a case of diaphragmatic and action myoclonus as the presenting feature of non-ketotic hyperglycemic state in a previously undiagnosed diabetic woman. Myoclonus as the presenting feature of diabetic amyotrophy has also been described [12,13]. Ocular flutter with myoclonus has been causally associated with hyperosmolar hyperglycemic state [14]; however, in that case, the patient had also uremia and subarachnoid hemorrhage [14]. Opsoclonus and myoclonus have also been reported in hyperosmolar hyperglycemic state [15]. Ghia et al [16] described a 17-month-old child who presented with opsoclonus-myoclonus-ataxia syndrome. Investigations revealed a thoracic neuroblastoma and eleven days later, she re-presented with DKA [16]. Our case is unique from all other previously published cases since myoclonus was the presenting manifestation of DKA in an adult patient with previously undiagnosed T1DM. As brain MRI showed no abnormality and functional neuroimaging and somatosensory evoked potential could not be done in our setup due to infrastructural limitations and COVID-19 regulations, exact localization (cortical or brainstem) and pathophysiological mechanism of myoclonus in this case remained unknown. In the present case, myoclonus could have occurred because of gamma-aminobutyric acid depletion that can be due to either hyperglycemia alone [2,8,9] or mediated by anti-GAD65 [17,18,19]. Anti-GAD-65 antibody, known to be pathophysiologically associated with a plethora of neurological manifestations, including myoclonus, probably did not play any role in our case as correction of hyperglycemia alone abated myoclonus obviating any need for steroids or immunotherapy [17]. Moreover, no recurrence of myoclonus in follow-up visits further strengthened this hypothesis. DKA-associated hyperviscosity, ketosis, vasculopathy and dyselectrolytemia may also act in synergism to cause hyperkinetic movements [20].

The major pitfalls while managing this case were the inability to correctly identify movement semiology and the inappropriate use of anti-epileptic drugs before ruling out common metabolic derangements that led to a delayed diagnosis.

## Conclusion

While acute movement disorders (mostly chorea and ballism) as the presenting feature of non-ketotic hyperglycemic complications (mostly among type-2 diabetics) are amply described in the literature, similar reports among DKA and T1DM patients are scarce [1,21]. Myoclonus as the presenting manifestation of DKA in a previously undiagnosed T1DM patient has never been previously reported. Thus, this case report certainly is an important addition to the literature describing various movement disorders among T1DM. Our case reiterates the importance of rapid bedside measurement of CBG among all patients presenting with acute onset abnormal movements irrespective of their past glycemic status. This simple yet life-saving approach can clinch the diagnosis at the earliest and thus will avoid costly investigations and mismanagement.
